# That’s not what you said the first time: A theoretical account of the relationship between consistency and accuracy of recall

**DOI:** 10.1186/s41235-016-0012-9

**Published:** 2016-11-05

**Authors:** Sarah E. Stanley, Aaron S. Benjamin

**Affiliations:** University of Illinois at Urbana-Champaign, 603 East Daniel Street, Champaign, IL 61820 USA

**Keywords:** Memory, Eyewitness, Accuracy, Output-bound accuracy, Inconsistent, Consistency, Reminiscence, Reminisced, Forgotten, Obliviscence, Multiple tests, Hypermnesia

## Abstract

Over multiple response opportunities, recall may be inconsistent. For example, an eyewitness may report information at trial that was not reported during initial questioning—a phenomenon called *reminiscence*. Such inconsistencies are often assumed by lawyers to be inaccurate and are sometimes interpreted as evidence of the general unreliability of the rememberer. In two experiments, we examined the output-bound accuracy of inconsistent memories and found that reminisced memories were indeed less accurate than memories that were reported consistently over multiple opportunities. However, reminisced memories were just as accurate as memories that were reported initially but not later, indicating that it is the *inconsistency* of recall, and not the later addition to the recall output, that predicts lower accuracy. Finally, rememberers who exhibited more inconsistent recall were less accurate overall, which, if confirmed by more ecologically valid studies, may indicate that the common legal assumption may be correct: Witnesses who provide inconsistent testimony provide generally less trustworthy information overall.

## Significance

The legal system often asks witnesses to describe an event on multiple occasions and relies on witnesses to recount events consistently each time they are asked. This expectation reflects a misguided perspective on the fragile nature of retrieval. In two experiments, we show that repeated attempts to recall a single list of items leads to many inconsistencies. Such inconsistently reported information is less accurate, a finding that is consistent with the expectations and behavior of lawyers in the courtroom. However, it does not matter whether such inconsistencies are reported on an early test and forgotten on a later one, or omitted on an early test and first introduced on a later one. This fact suggests that details introduced during trial should not be viewed with any greater suspicion than details reported before trial but forgotten during trial. Importantly, people who had more inconsistencies in their recall also exhibited lower accuracy even in the information that they consistently recalled, suggesting that less credence should be placed on an eyewitness’s entire account if they produce many inconsistencies. These results, taken together, indicate ways in which certain problems in eyewitness memory can be more profitably addressed with a combination of applied research that is superficially similar to the conditions under study and use-inspired, focused basic research that takes advantages of well-established paradigms and high levels of experimental control.

## Background

In the American justice system, witnesses to a crime are generally asked to repeat their account of an incident numerous times: to the police, in a disposition, in meetings with attorneys, and in court. When recounting a story so many times, and under such varied conditions, discrepancies are likely to occur. Such inconsistencies are often viewed as a sign of an unreliable and untrustworthy witness. Lawyers search for inconsistencies in witnesses’ accounts and use them to attack witnesses’ credibility (Alavi & Ahmad, 2002; Kerper, 1997), drawing their entire testimony into question. The technique is quite effective: A study using mock trials found that mock jurors exposed to inconsistent testimony found the eyewitness less effective and the defendant less culpable, and, as a consequence, they were less likely to convict (Berman & Cutler, 1996).

Despite the legal implications, surprisingly little research has been done to investigate the relationship between consistency and accuracy of recall. Basic memory research does not commonly focus on the form of accuracy relevant to this scenario, a form sometimes called *output*-*bound accuracy* (Koriat & Goldsmith, 1994). Output-bound accuracy is to be contrasted with the type of accuracy that is typically referred to in laboratory studies, which can be called *input*-*bound accuracy. Input*-*bound accuracy* refers to the proportion of studied items that are successfully recalled (i.e., the number of correctly recalled items divided by the number of things originally studied). This measure of memory is used in the vast majority of basic laboratory research on memory and places an emphasis on the amount of correct information reported about an event (Koriat & Goldsmith, 1994). In eyewitness literature, this is often referred to as *completeness*, as it reflects how thoroughly a witness describes an event. (This is the “whole truth” part of the famous oath used in U.S. courts.) *Output*-*bound accuracy* refers to the percentage of the items recalled that are correct (i.e., the number of correct things recalled divided by the total number of things recalled). This is the “nothing but the truth” part of the oath and is more relevant in circumstances where the specific details of the original event are unknown, such as the eyewitness scenario we have been relying on here. It reflects how much of a witness’s testimony is true. In this paper, when we talk about the accuracy of a memory, we mean specifically its output-bound accuracy.

Most of the relevant research on the relationship between consistency and accuracy can be found in the eyewitness memory literature. This research uses paradigms that, as we detail below, provide challenging circumstances under which to evaluate the accuracy of recall. It is thus perhaps not surprising that there are inconsistencies among those reports. The goal of the present research is to use the many advantages of very basic laboratory memory research to complement the small amount of applied work on this interesting problem.

An additional important distinction is related to the types of inconsistencies across multiple recall opportunities. Here we can distinguish between two general types: forgotten details, which are provided in an earlier account and omitted in a later one, and reminisced details, which are included in a later account but not mentioned in prior accounts.^1^ Note here that forgotten details are not ones that were never reported, but rather ones reported at least once and then omitted in later testimony. The focus of research on the consistency of recall has been on reminiscence, probably because forgetting but not reminiscence is compatible with the well-accepted notion that memory declines over time (Erdelyi, 2010).

Inconsistent recall could reflect a number of established psychological phenomena. One possibility is that rememberers may be more willing to report memories that they are unsure about on later opportunities than on earlier ones. Such a shift in reporting policy would result in new details or new memories being included in later testimony (cf. Koriat & Goldsmith, 1996). Another possibility is that exposure to postevent information from another source leads to changes in testimony (Johnson, Hashtroudi, & Lindsay, 1993), or that postevent information reminds the rememberer of forgotten aspects of the original event (Benjamin & Ross, 2011; Benjamin & Tullis, 2010; Tullis, Benjamin, & Ross, 2014). Yet another possibility is that reminiscence occurs simply because there is more cumulative retrieval time on later than on earlier retrieval attempts, an explanation Roediger and Thorpe (1978) proposed to account for hypermnesia (the enhancement of memory over multiple recall tests that is seen when reminiscence exceeds forgetting). None of these perspectives can also explain why details are sometimes reported and then omitted at a later date.

However, one general perspective, widely accepted in the memory literature, provides a straightforward explanation of both forgetting and reminiscence without reference to additional events or mechanisms. By that view, the success of any retrieval event is a product of both the cues available during retrieval and the ones experienced during encoding (Tulving & Thomson, 1973). In an eyewitness situation, the various retrieval attempts may take place under highly varying physical, mental, and emotional conditions, all of which can serve as retrieval cues. As retrieval cues change, details that were once recalled may become inaccessible and details that were inaccessible may be recalled (see also Fisher, Brewer, & Mitchell, 2009). Such fluctuation is a well-accepted theoretical mechanism for *spontaneous recovery* of previously extinguished associations in animal behavior, and can be straightforwardly applied to human recall as well (Estes, 1955; see also Bower, 1972).

If inconsistent recall reflects the fluctuation of cues across retrieval situations, then two effects should be apparent. First, details that are recalled across situations are more likely to be accurate than inconsistently recalled details, because those memories have proven themselves to be more robust to the variance of cues. Second, reminisced details should not be less likely to be accurate than forgotten ones. Any differences in accuracy between reminisced and forgotten details that have been reported in the literature may be due to a confounding with the elapsed time since the original encoding event for those two effects. Because cues are thought to fluctuate with time, an attempted retrieval at a more distant point is less likely to overlap with those present during encoding.

From the cue fluctuation perspective, reminisced memories differ from forgotten memories only insofar as they are first produced after a longer retention interval—the fact that they are produced after a failed attempt at retrieval gives them no special status. Consequently, both forgotten and reminisced details should be less accurate than consistently recalled details, but there is no reason to expect that they should be different from one another once the retention interval is controlled. It is for this reason that we take great care in our experiment to control for the retention interval.

We also seek to control for other sources of inconsistency in recall. Our use of the same free recall task across tests minimizes the possibility of shifts in reporting policy, as do our relatively short retention intervals. The introduction of postevent information is eliminated by keeping subjects in the laboratory between tests. Reminding is reduced by placing demanding but unrelated distractor tasks between all of the experimental events. We minimize the influence of total retrieval time by forcing subjects to recall for an extended period on each of the individual tests. As will become clear, inconsistencies are still ample under these conditions, suggesting that cue fluctuation may provide a more coherent explanation of the relevant phenomena.

A basic understanding of the relationship between accuracy and consistency is important in part because the American legal system tends to make assumptions about the accuracy of inconsistent witnesses. First, it is assumed that inconsistent details are inaccurate (Fisher et al., 2009). Second, it is assumed that some types of inaccuracies are worse than others. This is particularly true of reminisced memories, which lawyers are trained to use to discredit the witness in a process known in the legal community as “impeachment by omission” (McElhaney, 1987). This effect finds some support in the memory literature as well: Untrained observers expect reminisced memories to be much lower in accuracy than consistently produced information, and even to be lower in accuracy than they actually are (Oeberst, 2012). Trained detectives exhibit the same bias (Krix, Sauerland, Lorei, & Rispens, 2015). Third, it is assumed that inconsistent testimony is indicative of an unreliable witness—that is, that their entire testimony should be called into question, and not just the inconsistent parts. A fourth assumption follows from the third: that inconsistent testimony should be uncommon.

Three reports in the eyewitness memory literature bear on these issues directly (Brock, Fisher, & Cutler, 1999; Gilbert & Fisher, 2006; Oeberst, 2015). Gilbert and Fisher (2006) had subjects watch short mock crime videos; the subjects were then interviewed on two separate occasions. When comparing these two interviews, they found that inconsistencies were ubiquitous, with 98 % of all subjects reminiscing at least two details. On average, subjects recalled 20.4 details consistently, reminisced 8.4 details on the second test, and forgot 9.2 details from the first test to the second.^2^ Consistently recalled items were in fact significantly more accurate than forgotten items, which were in turn significantly more accurate than reminisced items; however, the accuracy of all the reported items was high. Brock and colleagues (1999) used a similar procedure: Subjects viewed a video of a traffic accident and were then interviewed twice (using the cognitive interview method; Fisher et al., 2009). They found that forgotten details were less likely to be accurate than consistently recalled details. (They did not examine reminisced details.) A third study using a video of a theft found that reminisced and forgotten details exhibited similar levels of accuracy, and were only slightly lower than those evidenced by consistent details (Krix et al., 2015). A similar pattern was reported by Oeberst (2015), evaluated memory for a live event in a classroom.

The use of an eyewitness task to evaluate memory consistency has costs and benefits. On the plus side, the generalization from these results to actual eyewitness memory is more straightforward than if a more traditional list-based laboratory memory task had been used. On the other hand, aspects of the procedure lead to questions about validity that might be more easily resolved in a list memory task and the controls that it affords. Most importantly, it is difficult to objectively define a “detail” in a report of a witnessed event. If a subject reports that a suspect wore pants and then later that the suspect wore jeans, is that a consistent report (because they reported the pants on both occasions) or a reminiscence (because there is an additional detail on the second report that the pants are made of denim)? Additionally, details may not be independent—remembering some details may allow for logical deductions about other details. Furthermore, not all of the details are of equal importance: Some may be trivial and others crucial. If a subject reports the presence of a blue sky, is it reasonable to consider that a relevant detail? All of these difficulties may be the source of some of the inconsistencies between the results in the prior literature—it is unclear from those papers, for example, whether forgotten details are more accurate than reminisced details or of equivalent accuracy. These problems are easily solved by moving to a list-based memory task, where the number and labels for the memoranda are clear and subjects know exactly what they are expected to report. Moreover, in a list-based task, items are independent and of roughly equal importance. This research is thus best considered to be use-inspired rather than applied. Our task does not “look like” an eyewitness memory event, but the basic findings may be relevant to those situations, especially if confirmed by a wider variety of designs. As with all such research, there may be factors involved in real-world eyewitness situations that limit the generalizability of the results of our work.

Only one experiment that we know of in the literature (Oeberst, 2012; Experiment 1) used a list-based procedure and examined the relationship between accuracy and consistency of recall. In that experiment, reminisced items were not found to be less accurate than consistently recalled items, a result inconsistent with all of the results from eyewitness paradigms reviewed earlier.

Our procedure uses a three-test design, which enables us to additionally evaluate whether an item reminisced early (say, during a deposition) is more likely to be accurate than an item reminisced later (say, during court). We know of no extant literature examining this question. Our experiments use a unique two-group, three-block procedure that enables control of key potential confounders in making this comparison.

A related question that follows from an investigation of accuracy and consistency concerns individual differences. Do people who report more inconsistent details exhibit lower accuracy for the consistently recalled material that they produce? Gilbert and Fisher (2006) found a small negative correlation that was not statistically significant relating the number of inconsistencies and the accuracy of the rest of the testimony. However, that analysis suffers from the same potential measurement difficulties discussed earlier—a fact that may make small correlations difficult to detect. Taken at face value, the lack of a statistically significant correlation would cast doubt on the commonly held belief that inconsistent witnesses are not trustworthy. Yet, the cue fluctuation perspective, combined with individual differences, would seem to predict that inconsistent recall should be related to lower accuracy among consistently recalled items. If subjects vary in their willingness to report a memory when the mnemonic evidence is uncertain (as we know they do; Koriat & Goldsmith, 1996), then some of the variance in the number of inconsistencies produced will reflect differences in a reporting criterion. Rememberers with a lower reporting criterion will exhibit overall lower accuracy (because of the addition of low-confidence reports to the protocol) and also produce more inconsistent details (because the exact content of those low-confidence reports will be more likely to vary with fluctuations in cues). Consequently, in contrast to the past research, we expect to see a positive relationship between accuracy and consistency across subjects.

In our experiments, we set out to evaluate three questions that follow directly from the prior literature and the cue fluctuation perspective:

1. Are inconsistently recalled items less accurate than consistent items?
2. Do reminisced and forgotten items exhibit similar levels of accuracy when the time of production is controlled?
3. Do people who demonstrate more inconsistent recall have lower overall accuracy, even for items that are recalled consistently?

We choose to evaluate these relationships using list-learning tasks. To this end, we had subjects study common items, thus rendering the scoring (almost) completely objective and eliminating the aforementioned concerns regarding evaluating recall of mock crimes.

One confound ubiquitous in prior research is the different production times for items that were reminisced or forgotten. By definition, in a two-test design, reminisced items are produced for the first time at a later delay from the study event than are forgotten items. In our experiments, we used a between-subjects design where both groups take multiple tests but the retention intervals are arranged such that one group takes its second test at the same lag at which the second group takes its first test. This procedure allows a comparison of reminisced items from one group with the forgotten and consistent items from another group, because the time of the original production is the same for reminisced, forgotten, and consistent details.

## Experiment 1

In this experiment, subjects studied a series of pictures of simple, nameable objects and were administered multiple tests of free recall in which to recall those objects.

### Methods

#### Subjects

The subjects were 87 University of Illinois at Urbana-Champaign undergraduate students participating in exchange for course credit. Ten participants had incomplete data due to computer problems and were excluded from the analysis.

#### Design

The experiment had a two-factor, quasi-experimental design. The first experimental factor was the number of tests. Group A (*n* = 40) took three free-recall tests with a short retention interval before the second test, and group B (*n* = 37) had two recall tests with a long retention interval. The second, quasi-experimental factor was response pattern (forgotten, reminisced, and consistent). The dependent variables were the number and output-bound accuracy of items recalled.

#### Materials

The stimuli included 60 simple, single-item colored drawings from Rossion and Pourtois (2001), available at http://spell.psychology.wustl.edu/Rossion_stimuli/. The images were displayed on a computer screen in a random order for 5 seconds, each with a 1-second interstimulus interval. A Tetris-like, tile-matching puzzle computer game was used as a distractor task after the study phase.

#### Procedure

**Fig. 1:**
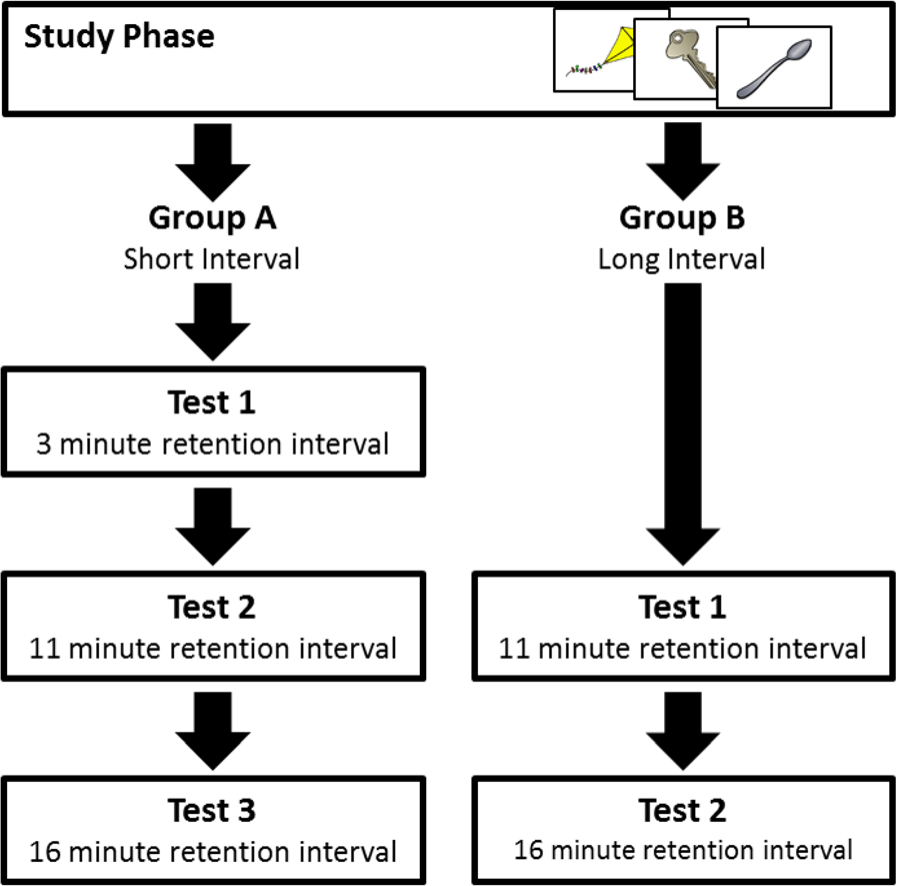
Study design for Experiments 1 and 2

Participants were instructed that they were going to be shown some images and that they should study the images and try to remember them for a later test. Participants then viewed all 60 images in a random order. As shown in Fig. 1, participants in Group A completed a 3-minute distraction interval, followed by Test 1, then another 3-minute distraction interval followed by Test 2, then another 3-minute distraction interval followed by Test 3. Participants in Group B had an 11-minute distraction interval, followed by Test 1 and then a 3-minute distraction interval followed by Test 2. All tests were identical other than abbreviated instructions for the second and third tests; instructions were self-paced with a minimum exposure of 20 seconds prior to the first test and 5 seconds prior to the subsequent tests. Each test was a 5-minute free recall test in which a subject was presented with 60 blanks on the screen and directed to type as many items as they could remember, in any order, using only 1 word to name each item. They were encouraged to spend the entire 5 minutes trying to remember and could not continue with the experiment until the 5 minutes had elapsed.

### Results and Discussion

Accuracy was assessed using both a within-subject and a between-subjects comparison. The within-subject comparison does not control for time of production, but the between-subjects comparison does. To ensure greater compatibility across the groups, the third test for Group A was ignored for all comparisons except those comparing reminiscence after one test or two; that is, an item was considered consistent if it was found on Test 1 and Test 2, regardless of whether it was found on Test 3. The accuracy and frequency for each response type are listed in Tables 1 and 2, respectively. The total number of responses on each test is shown in Table 3.

**Table 1:** Output-bound accuracy (SD) for consistent, reminisced, and forgotten items by time of test on which they were first output

Experiment 1	Group A	Group B	Groups A and B
	Short Interval	Long Interval	
Consistent	.89 (.16)	.92 (.10)	.91 (.13)
Forgotten	.74 (.32)	.72 (.34)	.73 (.33)
Reminisced (second test)	.69 (.34)	.75 (.27)	.72 (.31)
Reminisced (third test)	.67 (.42)		
Experiment 2	Group A	Group B	Groups A and B
	Short Interval	Long Interval	
Consistent	.87 (.10)	.86 (.14)	.87 (.12)
Forgotten	.65 (.39)	.61 (.38)	.63 (.38)
Reminisced (second test)	.71 (.31)	.66 (.29)	.69 (.30)
Reminisced (third test)	.54 (.41)

**Table 2:** Average frequency (SD) for consistent, reminisced, and forgotten items by time of test on which they were first output

Experiment 1	Group A	Group B	Groups A and B
	Short Interval	Long Interval	
Consistent	19.60 (6.55)	22.05 (7.09)	20.78 (6.88)
Forgotten	4.13 (6.74)	2.89 (2.42)	3.53 (5.15)
Reminisced (second test)	5.60 (7.21)	5.68 (4.78)	5.63 (6.12)
Reminisced (third test)	3.60 (6.26)		
Experiment 2	Group A	Group B	Groups A and B
	Short Interval	Long Interval	
Consistent	20.55 (6.60)	19.05 (6.49)	19.81 (6.57)
Forgotten	2.72 (1.82)	2.89 (2.42)	2.80 (2.13)
Reminisced (second test)	3.73 (2.77)	3.82 (3.11)	3.78 (2.94)
Reminisced (third test)	1.97 (1.74)

**Table 3:** Average number of responses (SD) for each test

Experiment 1	Test 1	Test 2	Test 3
Group A (short interval)	23.73 (9.68)	25.20 (10.54)	22.75 (9.12)
Group B (long interval)	24.96 (7.80)	27.73 (9.27)	
Experiment 2	Test 1	Test 2	Test 3
Group A (short interval)	23.28 (6.52)	24.29 (6.32)	22.32 (6.39)
Group B (long interval)	21.93 (6.16)	22.89 (6.54)	

All results were interpreted using both a strict grading standard and a lenient grading standard. The strict grading standard was fully automated and required subjects to type the exact name of each item, as decided by the experimenter prior to the experiment. The lenient grading standard was partially automated. Words that were deemed incorrect by the strict standard were interpreted by an experimenter blind to condition, who allowed for minor misspellings, confusion of plural and singular, and the use of synonyms. Any corrections were automatically applied to other errors, ensuring reliability across subjects. The results were similar for both grading schemes, so only the more objective, strict grading standard is reported. All statistical tests yielded the same conclusions in both grading schemes unless stated otherwise.

#### Within-subject effect of response consistency on output-bound accuracy

**Fig. 2:**
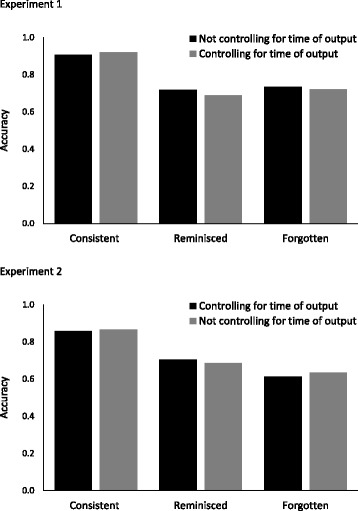
Output-bound accuracy as a function of response pattern. The *black bars* are from the within-subject contrast, and the *gray bars* are from the between-subjects contrast

This analysis lumps together Group A and Group B and does not control for time of production. As can be seen in Fig. 2, consistently recalled items exhibited a higher degree of output-bound accuracy than either forgotten (*t* [64] = 4.50, *p* < .001) or reminisced (*t* [67] = 5.69, *p* < .001) items. The degrees of freedom were reduced because not all subjects produced items in each response pattern. The difference in accuracy between forgotten and reminisced items was not significant (*t* [57] = 0.22, *p* = .83).

#### Between-subjects effect of response consistency on output-bound accuracy

Some of these comparisons can be made on a between-subjects basis, which allows us to control for the time of first output when comparing the accuracy of reminisced items from Group A with consistently recalled and forgotten items from Group B. In the between-subjects comparison, consistently recalled items again revealed higher accuracy than reminisced items (*t* [38.09] = 3.83, *p* < .001). The difference in accuracy between forgotten and reminisced items was again not significant (*t* [62.25] = 0.37, *p* = 0.71).

#### Inconsistencies in recall and overall subject accuracy

**Fig. 3:**
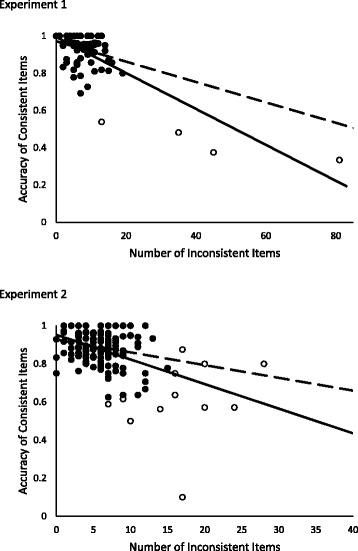
Accuracy of subject’s consistent items as a function of the number of inconsistently recalled items. The *dashed line* represents the best-fit regression line when the outliers (marked as *circles*) are excluded. The *solid line* represents all data

A final comparison evaluates whether the inconsistency of subjects’ recall reveals anything about the accuracy of their consistently recalled material. As shown in Fig. 3, there was a negative correlation between the number of inconsistent items produced across the first two tests and the accuracy of the consistently produced items (*r* = −.76, *t* [75] = −10.27, *p* < .001). The correlation remains even when outliers (more than 2 SD away from the mean number of inconsistent items or the accuracy) are excluded (*r* = −.30, *t* [71] = −2.65, *p* = .01).

## Experiment 2

To evaluate the robustness of the results of Experiment 1, we conducted another experiment with a slightly more complex encoding scenario. This experiment also provides an opportunity to evaluate the accuracy of early versus late reminiscence with somewhat greater power, as we detail below. In this experiment, we moved toward more complicated stimuli by using real-world objects viewed at once rather than a series of images on a computer. This procedure represents a modest increase in external validity by having items in a physical configuration in which people can see them from different angles, move closer and further away, examine details present in real objects, and allocate their attention according to their motivation. The stimuli still have the advantage of constituting a set of nameable objects that subjects can easily produce on a verbal test.

### Methods

#### Subjects

Subjects were 150 University of Illinois at Urbana-Champaign undergraduate students participating in exchange for course credit.

#### Design

The design and variables were the same as those in Experiment 1. Group A (*n* = 76) received three free recall tests, and Group B (*n* = 74) received two recall tests.

#### Materials

The stimuli included 40 common items arranged on a table (see Appendix 1 for a photograph). The arrangement was the same for all subjects. The items were a combination of objects that were chosen to be easy to name. The distractor task and test administration were the same as those in Experiment 1.

#### Procedure

Subjects were brought into the laboratory one at a time. Before entering the room, subjects were told that there were assorted objects arranged on a table and that their task was to study the items on the table for 5 minutes. They were told they could move around the room, but they were asked not to touch anything. Participants were then left alone in the room for approximately 5 minutes to study the items. After the study period, subjects were directed to an adjacent room where they were to complete the distractor and test phases on a computer. The rest of the procedure was identical to that in Experiment 1.

### Results and Discussion

We conducted all the same analyses as those used in Experiment 1. The accuracy for each response type is listed in Table 1. The total number of responses on each test is shown in Table 3.

#### Within-subject effect of response consistency on output-bound accuracy

As can be seen in Fig. 2, consistent items exhibited a higher degree of output-bound accuracy than either forgotten (*t* [136] = 7.29, *p* < .001) or reminisced (*t* [139] = 7.41, *p* < .001) items. The difference in accuracy between forgotten and reminisced items was not significant (*t* [131] = 1.06, *p* = .29).

#### Between-subjects effect of response consistency on output-bound accuracy

In the between-subjects comparison (which allows us to control for the time of output), consistently recalled items revealed higher accuracy than reminisced items (*t* [103.67] = 3.89, *p* < .001). The difference in accuracy between forgotten and reminisced items was not significant (*t* [124.01] = 1.58, *p* = .12).

#### Inconsistencies in recall and overall subject accuracy

As shown in Fig. 3, there was a negative correlation between the number of inconsistent items produced across the first two tests and the accuracy of the consistently produced items (*r* = −.46, *t* [147] = −6.26, *p* < .001). The correlation remains even when outliers (more than 2 SD away from the mean number of inconsistent items or the accuracy) are excluded (*r* = −.23; *t* [135] = −2.75, *p* = .01).^3^

## Combined analysis of experiments 1 and 2

Because one of our central findings is a lack of an effect (between reminisced and forgotten items), we conducted all of the reported analyses again using pooled data from both experiments, and we assessed power for each of the relevant comparisons.

### Within-subject effect of response consistency on output-bound accuracy

Consistently recalled items exhibited a higher degree of output-bound accuracy than forgotten items (*t* [201] = 8.54, *p* < .001). Using Hedges’s *g*_av_, as suggested by Lakens (2013) for use with correlated measures, we found this to be a large effect size (*g*_av_ = 0.82). Consistent items also exhibited a higher degree of output-bound accuracy than reminisced items (*t* [207] = 9.34, *p* < .001). This was also a large effect (*g*_av_ = 0.84). Despite high power (.98) to detect a small-to-medium effect (0.3), no significant difference between reminisced and forgotten items was found (*t* [189] = 0.85, *p* = 0.40).

### Between-subjects effect of response consistency on output-bound accuracy

In the between-subjects comparison (which allows us to control for the time of output), consistently recalled items revealed higher accuracy than reminisced items (*t* [143.03] = 5.44, *p* < .001). Using Hedges’s *g*_s_ (as recommended for independent data; Lakens [2013]), we found this to be a medium-to-large effect size (*g*_s_ = 0.73). The difference in accuracy between forgotten and reminisced items was not significant (*t* [188.75] = 1.10, *p* = .27), though it should be noted that the power to detect a small-to-medium effect (0.3) was only middling (.57).

### Inconsistencies in Recall and Overall Subject Accuracy

There was a negative correlation between the number of inconsistent items produced across the first two tests and the accuracy of the consistently produced items (*r* = −.55, *t* [224] = −9.77, *p* < .001). The correlation remains even when outliers (more than 2 SD away from the mean number of inconsistent items or the accuracy) are excluded (*r* = −.25, *t* [212] = −3.69, *p* < .001).

### Time of initial output and accuracy of reminisced items

One additional question about reminiscence concerns the question whether an item reminisced on a later test is less likely to be accurate than an item reminisced on an earlier test. A post hoc, within-subject comparison revealed that items first produced on a second test were significantly more accurate than those produced on a third test (*t* [95] = 2.38, *p* = .02).^4^^,^ 5 The effect size (*g*_av_ = 0.31) suggests a small-to-medium practical significance.

## Conclusions

The general view that inconsistencies in recall reflect the fluctuation of cues across retrieval situations provides a simple perspective from which to relate the accuracy and consistency of recalled information. We sought two specific effects here. First, consistently recalled details should be more accurate than inconsistently recalled details, because those memories have proven themselves to be more robust to the variance of cues across retrieval conditions. Second, reminisced details—ones produced in later but not earlier retrieval attempts—should be no less accurate than forgotten ones, produced in earlier but not later attempts. Both of these predictions were confirmed and are consistent with some prior work using eyewitness paradigms (Krix et al., 2015; Oeberst, 2015). The work reported here is the first that makes these comparisons under conditions in which retention interval is deconfounded from response type.

Additionally, we found that items reminisced on an earlier test were significantly more accurate than those reminisced on a later test. However, because of generally low power in those between-subjects contrasts, the effect of time on the accuracy of reminiscence can only be considered provisional and awaits further evidence.

Finally, we also found across both our experiments that the more inconsistencies a person had, the less accurate the person’s consistently produced items were. That is, there was a correlation between the number of inconsistent items that a subject produced and the accuracy of the individual’s consistently recalled items. Although this result appears inconsistent with the findings of Gilbert and Fisher (2006), the magnitude of the correlation between overall accuracy and number of reminisced details in their work (*r* = −.15) is comparable to the magnitude of our correlations when outliers are excluded.

In the theoretical picture provided here, the overlap between cues present at retrieval and those cues encoded at the time of the original event determines the likelihood of recall. Inconsistencies arise because of random fluctuations (and maybe nonrandom ones as well) with time and intervening events. Individual differences in the level of evidence that a rememberer demands prior to producing a memory yield the relationship between accuracy and consistency at the subject level.

Of course, the overlap in cues in our work is an entirely theoretical entity. There is no specification of the nature of these cues or what causes them to fluctuate (cf. Divis & Benjamin, 2014). Yet, it is interesting to consider what sorts of operational variables might reflect this overlap. One possibility is that self-reported confidence could be used as a measure. It is already known that confidence reflects a metamnemonic assessment of accuracy, is meaningfully related to accuracy, and determines the probability of reporting (Goldsmith & Koriat, 2008). If confidence is taken as a measure of cue overlap, then it should mediate the relationship between accuracy and consistency (see Odinot & Wolters, 2006). Indeed, confidence appears to be lower for inconsistently produced than for consistently produced details in an eyewitness report (Odinot, Wolters, & van Koppen, 2009), though its mediating role is unclear from that work. Given the central importance of confidence in other arenas of eyewitness behavior (Wixted, Mickes, Clark, Gronlund, & Roediger, 2015), this would seem to be a fruitful avenue for future research.

### Consistency of recall and the legal system

Witnesses are often asked to describe a witnessed event on multiple occasions ranging over a wide period of time and a variety of circumstances. The legal system relies on witnesses to recount events consistently each time they are asked, but this reflects a misguided perspective on the nature of human memory. The critical dependence of memory on contextual cues, many of which are not under the rememberer’s control, suggests that inconsistencies across circumstances should be common. Here we show that repeated attempts to recall a set of items lead to many inconsistencies and that such inconsistently produced information is less accurate than consistently produced information. This finding is compatible with the behavior of lawyers who argue for the unreliability of inconsistently produced information. We also found that people who had more inconsistencies in their recall were less accurate overall. This is an important finding that deserves verification across a wider range of study and testing circumstances. If these findings are replicated across a variety of different procedures, including ones that use eyewitness memory tasks, it would suggest that less weight should be placed on an eyewitness’s account if the eyewitness produces many inconsistencies. This result is also consistent with the behavior of lawyers who use inconsistencies to more generally impeach a witness’s credibility. However, the ubiquity of inconsistencies must be kept in mind—it is only those eyewitnesses who exhibit a tendency for excessive amounts of inconsistent recall who should be regarded with suspicion.

It is important to acknowledge certain limitations in attempting to generalize the results of this research to true eyewitness scenarios. Our research involved only people who have no incentive to lie; there is very little pressure to provide answers and no penalty for providing incorrect answers; retention intervals are short; and our tests are strictly free-recall with no opportunity for subjects to gain additional information. Additionally, as discussed previously, real eyewitnesses report on events, not on lists.

Our research, as well as that of others (Gilbert & Fisher, 2006; Krix et al., 2015; Oeberst, 2012, 2015), does suggest that inconsistencies are common among people with no incentive to lie; as such, small or few inconsistencies should not be considered an indication of dishonesty or general inaccuracy. However, because we have not measured inconsistencies within subjects with a motivation to lie it remains possible that higher levels of inconsistency may, in fact, be indicative of lying.

In addition, there appears to be no reason to view reminisced details with especial suspicion. Reminisced information appears to be comparable in accuracy to forgotten information, indicating that the court’s particular distrust of reminisced items may be unfounded. Inconsistent details should be accorded less trust, but the nature of the inconsistency does not appear to matter.

## Figures and Tables

**Fig. 4:**
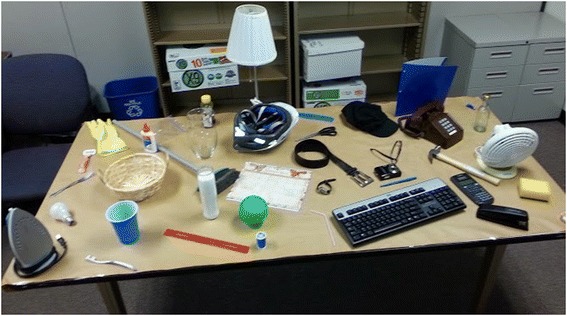
Display used for Experiment 2

## Footnotes

1 A third type of inconsistency, contradiction, in which details change across accounts, is not addressed separately here.

2 These numbers were obtained by averaging across the conditions listed on the left side of Table 2 (p. 732) and were weighted by the number of participants per condition listed in Table 1 (p. 730; Gilbert & Fisher, 2006).

3 When lenient grading was used, the correlation was significant before removing outliers (*r* = −.30, *t* [147] = 3.86, *p* < .001) but was not significant when outliers more than 2 SD away from the mean were excluded (*r* = −0.15, *t* [136] = 1.78, *p* = .08)

4 The within-subject comparison offers high power (.83) but confounds the time of original output with the number of failed retrieval attempts prior to being output. This can be deconfounded with two between-subjects comparisons which revealed that items first output on an earlier test were significantly more accurate than those output first on a later test (*t* [175.02] = 2.08, *p* = .04); however, the accuracy of reminisced items did not depend on when an item was first output (*t* [205.93] = 0.23, *p* = .82). The power in these comparisons to detect a medium effect was middling (.58 and .56, respectively).

5 This difference was not statistically significant when lenient grading was used (*t* [76] = 1.29, *p* = .20).
